# Comparative study on saponin fractions from *Panax notoginseng *inhibiting inflammation-induced endothelial adhesion molecule expression and monocyte adhesion

**DOI:** 10.1186/1749-8546-6-37

**Published:** 2011-10-13

**Authors:** Nan Wang, Jian-Bo Wan, Shun-Wan Chan, Yan-Hui Deng, Nan Yu, Qing-Wen Zhang, Yi-Tao Wang, Simon Ming-Yuen Lee

**Affiliations:** 1 State Key Laboratory of Quality Research in Chinese Medicine (University of Macau), Macao SAR, China; 2 Institute of Chinese Medical Sciences, University of Macau, Macao SAR, China; 3 Translational Medicine R&D Center, Shenzhen Institutes of Advanced Technology, Chinese Academy of Sciences, Shenzhen, 518000, China; 4 State Key Laboratory of Chinese Medicine and Molecular Pharmacology, Department of Applied Biology and Chemical Technology, Hong Kong Polytechnic University, Hong Kong SAR, China

## Abstract

**Background:**

*Panax notoginseng *is commonly used for the treatment of cardiovascular diseases in China. The present study investigates the effects of three different saponin fractions (*ie *total saponins, PNS; protopanaxadiol-type saponin, PDS; and protopanaxatriol-type saponin, PTS) and two major individual ingredients (*ie *ginsenoside Rg_1 _and Rb_1_) from *P. notoginseng *on the endothelial inflammatory response *in vitro *and *in vivo*.

**Methods:**

Recombinant human tumor necrosis factor-α (TNF-α) was added to the culture medium of human coronary artery endothelial cells (HCAECs) to induce an inflammatory response. A cell adhesion assay was used to determine the effect of the *P. notoginseng *saponin fractions on endothelial-monocyte interaction. The cell adhesion molecule (CAMs) expression, including ICAM-1 and VCAM-1, in the protein level on the surface of endothelial cells were measured by cellular ELISA. CAMs expression in mRNA level was also assayed by qRT-PCR in the HCAECs and the aorta of rat fed with high cholesterol diet (HCD). Western blotting was used to detect effect of the saponin fractions on CAMs protein expression in HCAECs. In addition, nuclear translocation of p65, a surrogate marker for NF-κB activation, was measured by immunostaining.

**Results:**

Three saponin fractions and two individual ginsenosides exhibited the inhibitory effects on monocyte adhesion on TNF-α-activated HCAECs and expression of ICAM-1 and VCAM-1 at both mRNA and protein levels *in vitro*. The saponin fractions exhibited a similar trend of the inhibitory effects on the mRNA expression of CAMs in the aorta of HCD-fed rat *in vivo*. These inhibitory effect of saponin fractions maybe attribute partially to the suppression of the TNF-α-induced NF-κB activation.

**Conclusion:**

Our data demonstrate that saponin fractions (*ie *PNS, PDS and PTS) and major individual ginsenosides (*ie *Rg_1 _and Rb_1_) have potential anti-atherogenic effects. Among the tested saponin fractions, PDS is the most potent saponin fraction against TNF-α-induced monocyte adhesion as well as the expression of adhesion molecules *in vitro *and *in vivo*.

## Background

Atherosclerosis (AS), a progressive disease characterized by the accumulation of lipids and fibrous elements in the large arteries, is the cause of most human heart diseases and strokes [1]. The role of vascular inflammation in atherosclerosis has been increasingly recognized in the past decade [2,3]. The early phase of vascular inflammation involves the recruitment of inflammatory monocytes from the circulation into the sub-endothelium, where they ingest lipid and become foam cells. This process is mediated predominantly by adhesion molecules, such as intercellular adhesion molecule-1 (ICAM-1) and vascular cell adhesion molecule-1 (VCAM-1) on the surface of vascular endothelium. Up-regulation of these adhesion molecules on endothelial cells is important in the initial stage of the inflammatory response in atherosclerosis [3,4]. Much interest is now focused on the determination of the therapeutic value of the inhibitors of endothelium-leukocyte adhesion.

The extract of *Panax notoginseng *has long been prescribed for the treatment of coronary heart diseases in China [5]. We recently showed that the total saponins from *P. notoginseng *(PNS) dramatically reduced the extent of atherosclerotic lesion in apolipoprotein E (Apo E)-deficient mice and that effect was associated with an anti-vascular inflammatory activity [6]. PNS is a chemical mixture containing more than 50 different saponins [5] and are classified into two main groups, namely the 20(S)-protopanaxatriol saponins (PTS), such as ginsenoside Rg_1_, and the 20(S)-protopanaxadiol saponins (PDS), such as ginsenoside Rb_1 _[5,7]. PDS and PTS showed diverse or even antagonistic pharmacological activities [8-11]; however, the active chemical component(s) in the PNS fraction responsible for the anti-vascular inflammation and the underlying molecular mechanism are largely unknown.

This study examines the anti-vascular inflammatory effects of three saponin fractions and two individual ginsenosides on the TNF-α-activated human coronary artery endothelial cells (HCAECs). The anti-vascular inflammatory action of the three saponin fractions is further evaluated by determining the mRNA expression of cell adhesion molecules (CAMs) in the aorta of high-cholesterol diet (HCD)-fed rats *in vivo*.

## Methods

### Quality control of chemical fractions

PNS (> 95% pure) was purchased from Wanfang Natural Pharmaceutical Company (China). In our laboratory, PTS and PDS were previously separated from PNS by DS-401 macroporous resins eluted with 30% and 80% (v/v) aqueous ethanol solutions respectively [7]. Ginsenosides Rb_1 _and Rg_1 _were purchased from the National Institute for the Control of Pharmaceutical and Biological Products (China). To ensure the consistency of efficacy, we determined the chemical characteristics of these fractions, including PNS, PTS and PDS using HPLC-UV. An Aglient 1100 series HPLC apparatus (USA) was operated under optimized conditions [12,13]. HPLC-grade acetonitrile was purchased from Merck (Germany). De-ionized water was prepared by a Milli-Q purification system (USA).

### Animals and treatment

Male Sprague-Dawley rats (170 ± 10 g), purchased from Guangdong Provincial Medical Laboratory Animal Center (China), were maintained on a 12-hour dark/light cycle in air-conditioned rooms (25 ± 2°C, 50 ± 5% humidity) with access to food and water *ad libitum*. After acclimation for one week, the rats were randomly assigned to nine groups (*n *= 8 per group). Group 1 (control) was fed a standard rat chow (~14% protein, ~10% fat and ~76% carbohydrate); groups 2-9 (treatment) were fed HCD, a standard rat chow supplemented with 1% cholic acid, 2% pure cholesterol and 5.5% oil. HCD-treated groups were gavage once every morning for 28 days with the vehicle, simvastatin (3 mg/kg), PNS (30 and 100 mg/kg), PDS (30 and 100 mg/kg) and PTS (30 and 100 mg/kg). At the end of the feeding, the rats were fasted overnight and sacrificed by cervical dislocation. Blood, liver and aorta were collected for analysis. This study was conducted according to protocols approved by the Ethics Committee of Hong Kong Polytechnic University.

### Cell culture and treatment

HCAECs (Cambrex, USA) were cultured in EGM-2 MV medium supplemented with SingleQuots kit (Cambrex, USA), including hydrocortisone, hFGF, VEGF, IGF-1, ascorbic acid, hEGF, R^3^-IGF-1, gentamicin/amphotericin-B, and 5% fetal bovine serum, at 37°C in a humidified 5% CO_2 _atmosphere. Cells with 85-90% confluence from passages two to six were used for the experiments.

PNS, PDS and PTS stocks of 1 mg/ml as well as Rb_1 _and Rg_1 _stocks (1 μM) were dissolved in Milli-Q water. The solutions were filtered through an Econofilter (0.22 μm, Agilent Technologies, USA). The samples were added to cultured cells at different final concentrations and incubated for 24 hours. To initiate an inflammatory response, we added 10 ng/ml recombinant human tumor necrosis factor-α (TNF-α; expressed in *Escherichia coli*, Sigma, USA) to the medium. The mixture was incubated with endothelial cells for four hours. Pyrrolidine dithiocarbamate (PDTC, purity > 99.0%; Sigma, USA) was used as positive control and incubated for two hours.

### Cell adhesion assay

Monocyte adhesion was determined by the starved THP-1 cells labeled with fluorescent dye Calcein-AM. HCAECs (5 × 10^3 ^cells/well) were plated in 96-well plates pretreated with various concentrations of different samples, and subsequently stimulated with 10 ng/ml TNF-α for four hours. Calcein-AM-labeled THP-1 cells (5 × 10^3 ^cells/well) and TNF-α-activated HCAECs were incubated together for 30 minutes. The total fluorescence intensity of each well was measured in a multi-well plate reader (Wallac 1420, Germany) with excitation at 485 nm and emission at 530 nm. Cells were then washed with phosphate-buffered saline three times to remove excess excess calcein-AM-labeled THP-1 cells. The measurement was repeated.

### Cellular ELISA assay

Cellular ELISA, modified from Rothlein [14], was used to measure the expression of ICAM-1 and VCAM-1 on the surface of endothelial cells. Briefly, HCAECs grown to confluence in a 96-well plate were treated with different samples followed by stimulation with TNF-α (10 ng/ml). After fixation and blocking, cells were incubated with anti-ICAM-1 (1:500) or anti-VCAM-1 (1:300) mAb for one hour, then with horseradish peroxidase-conjugated goat anti-mouse IgG at VCAM-1 (1:200) or ICAM-1 (1:400) respectively. Cells were exposed to the peroxidase substrate, and absorbance at 490 nm was measured in a fluorescence multi-well plate reader.

### qRT-PCR analysis

Total RNA was extracted from HCAECs with RNeasy mini kit (Qiagen, USA). SuperScript III^® ^First-strand synthesis system for real time RT-PCR (Invitrogen, USA) was used to reverse-transcribe and amplify the mRNA (0.7 μg) from each sample into cDNA. Oligonucleotide primers and TaqMan^® ^probes for human GAPDH, ICAM-1 and VCAM-1 were purchased from Applied Biosystems (USA). TaqMan^® ^universal PCR master mix (Applied Biosystems, USA) was used for quantitative assay. Real time PCR was performed on an ABI PRISM 7500 Sequence Detection System (Applied Biosystems, USA). All samples were assayed in triplicates and normalized on the basis of their GAPDH content.

At the end of the feeding, rats were sacrificed and the thoracic aorta (~15 mm) was rapidly dissected and placed into Tyrode's solution (NaCl 118 mM, KCl 4.7 mM, KH_2_PO_4 _1.2 mM, NaHCO_3 _25 mM, glucose 11 mM, CaCl_2 _2.5 mM, MgSO_4 _1.2 mM) at 4°C. The fat and connective tissue adhering to the adventitia were carefully cleaned from the aorta as much as possible with surgical scissors under a dissecting microscope. The total RNA of an isolated aorta was extracted with TRIzol reagent (Invitrogen, USA) according to the manufacturer's protocols. The same amount of RNA (4.0 μg) was reverse-transcribed and amplified into cDNA with a RevertAid™ first strand synthesis kit (Fermentas, Canada). Primers for the genes of interest were synthesized by Shanghai Gene Core BioTechnologies, China (Table 1). Real-time PCR was carried out with iQ™ SYBR^® ^Green SuperMix (Bio-Rad, USA) and normalized to GAPDH content.

**Table 1:** Primer sequences used for quantitative real-time polymerase chain reaction

gene name	Forward primer (5' to 3')	Reverse primer (5' to 3')	PCR product size (bp)	GeneBank **accession no**.
ICAM-1	AGACACAAGCAAGAGAAGAA	GAGAAGCCCAAACCCGTATG	234	NM_012967.1
VCAM-1	GGAGCCTGTCAGTTTTGAGAATG	TTGGGGAAAGAGTAGATGTCCAC	105	NM_012889.1
GAPDH	TGCACCACCAACTGCTTAG	AGTGGATGCAGGGATGATGT	180	NM_017008

### Western blotting

HCAECs (50 × 10^4 ^cells/dish) grown to confluence in a dish were pretreated with various concentrations of PNS, PDS and PTS and stimulated with 10 ng/ml TNF-α in 0.5% FBS medium for six hours. Cell pellets were lysed in RIPA lysis buffer (USA) with 1% PMSF, 1% protease inhibitor cocktail and 1% sodium orthovanadate. After treatment on ice for 30 minutes, cell lysates were centrifuged (Beckman Coulter, USA) at 11, 419 × g for 30 minutes at 4°C to remove cell debris; the protein content was measured with a BSA protein assay kit (Pierce, USA). The aliquot lysates were subjected to 10% SDS-PAGE (with 5% stacking gel) and transferred to a PVDF membrane (Bio-Rad, USA). The membrane was probed with mouse monoclonal antibody (mAb) against ICAM-1 (1:1000) and VCAM-1 (1:500) followed by horseradish peroxidase-conjugated secondary antibodies diluted 1:7500 and 1:2000 respectively and visualized with an ECL advanced western blotting detection kit (Amersham, UK) according to the manufacturer's protocol. Densitometric measurements of band intensity in the Western blots were performed using Quantity One software (Bio-Rad, USA).

### Immunofluorescence staining

HCAECs were cultured in a 24-well plate. After fixed with 80% ethanol for 10 minutes, the cells were incubated with monoclonal antibody against p65 (1:100) for one hour at room temperature, followed by incubation with anti-mouse IgG Alexa 488 antibody (1:100) for 30 minutes. After washed with PBS for three times, the cells were mixed with propidium iodide (1:1000) for ten minutes and finally were examined and photographed with a fluorescence microscope.

### Statistical analysis

All values were expressed as mean ± SD. Differences between groups were assessed by one-way analysis of variance (ANOVA) with SPSS for Windows (version 15, USA). The level of statistical significance was set at *P *< 0.05.

## Results

### Chemical characteristics of the tested fractions

**Figure 1:**
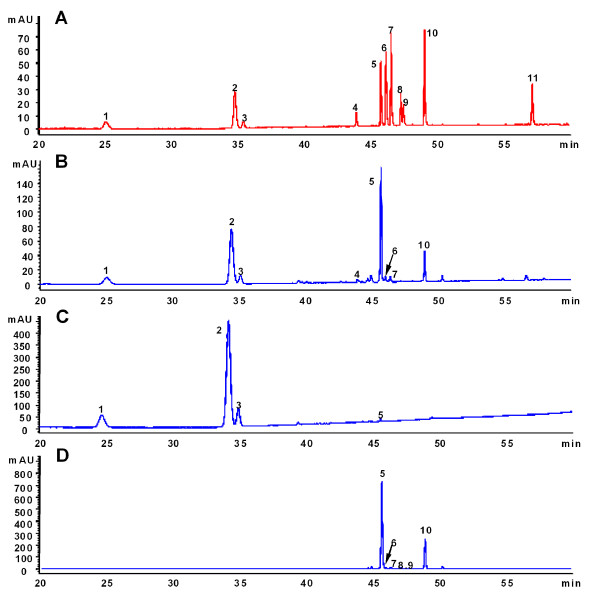
**HPLC-UV profiles of mixed standards (A), PNS (B), PTS (C), PDS (D) Peaks: 1, notoginsenoside R_1_; 2-11, ginsenoside Rg_1_, Re, Rf, Rb_1_, Rg_2_, Rc, Rb_2_, Rb_3_, Rd and notoginsenoside K**.

**Figure 2:**
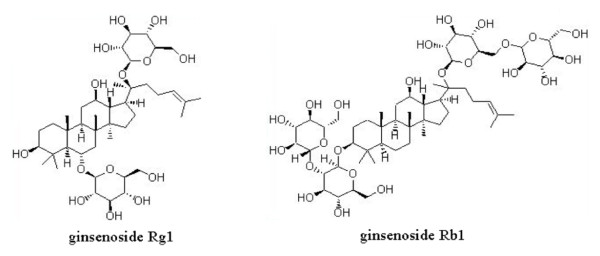
**Chemical structures of ginsenosides Rg_1 _and Rb_1_**.

The chemical characteristics of three fractions were determined to ensure quality consistency and standardization. Under optimized chromatographic conditions [7], the peaks corresponding to 11 chemical standards of different saponins were well separated and identified in 60 minutes (Figure 1A). Five compounds, namely notoginsenoside R_1_, ginsenosides Rg_1_, Re, Rb_1 _and Rd, were clearly identified as the major components of PNS (Figure 1B) and constituted approximately 90.2% of the total chemical composition of PNS. Among them, the first three saponins (*ie *notoginsenoside R_1_, ginsenoside Rg_1 _and Re) were the main components of the PTS fraction (Figure 1C) whereas Rb_1 _and Rd were the major components of the PDS fraction (Figure 1D). These compounds constituted approximately 88.2% and 92.6% of the total chemical composition of the PTS and PDS fractions respectively. Figure 2 shows the chemical structures of ginsenosides Rg_1 _and Rb_1 _from *P. notoginseng*.

### Saponins inhibit monocyte adhesion on activated endothelium

**Figure 3:**
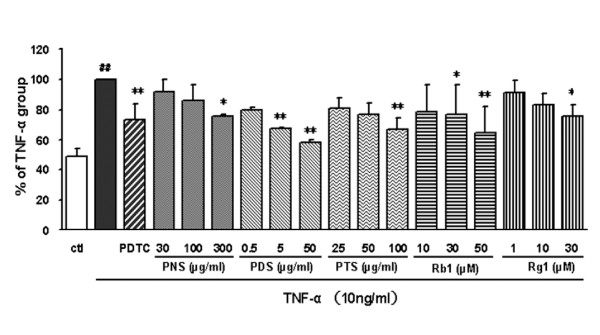
**Effects of fractions and ginsenosides from *P. notoginseng *on THP-1 cells adhesion to TNF-α-activated HCAECs**. PDTC was used as positive control. Bars represent mean ± SD (*n *= 3). ^## ^*P *< 0.01 vs. control group; * *P *< 0.05 and ** *P *< 0.01 vs. TNF-α group.

In order to identify which type of saponin was responsible for the anti-atherogenic effect of the PNS fraction *in vivo *[6], we compared saponin fractions (*ie *PNS, PTS and PDS) and the ginsenosides (Rg_1 _and Rb_1_) for inhibitory activity on THP-1 cells adhered to TNF-α-activated HCAECs, mimicking an early step of the pathogenesis of atherosclerosis. A low level of adherence of monocytes to unstimulated HCAECs was increased two-fold upon stimulation with TNF-α (Figure 3). The PDTC (10 μg/ml) positive control greatly reduced the adhesiveness of THP-1. Endothelial cells pretreated with each of the different samples of *P. notoginseng *exhibited dose-dependent but different inhibitory effect on the TNF-α-induced adhesion of monocytes to endothelial cells (Figure 3). After treatment with the PNS (300 μg/ml), PDS (50 μg/ml) and PTS (100 μg/ml) fractions, the monocyte-endothelial cell adhesion was reduced by 24.6%, 41.9% and 32.8% respectively. Comparison of the effective dose ranges and corresponding relative inhibition rates showed that the inhibitory effect of the PDS fraction on the adhesion of THP-1 cells to TNF-α-stimulated HCAECs was more potent than that of the PTS or PNS fraction. In addition, Rb_1 _and Rg_1 _significantly and dose-dependently inhibited the adhesion of THP-1 monocyte cells to TNF-α-stimulated HCAECs; Rb_1 _(50 μM, 55.5 μg/ml) and Rg_1 _(30 μM, 24 μg/ml) decreased the adhesion by about 35% and 24% respectively. In short, the trend of the inhibitory actions in this *in vitro *assay was that PDS was more effective than PTS which was more effective than PNS.

### Saponins inhibit the expression of TNF-α-induced endothelial adhesion molecules

**Figure 4:**
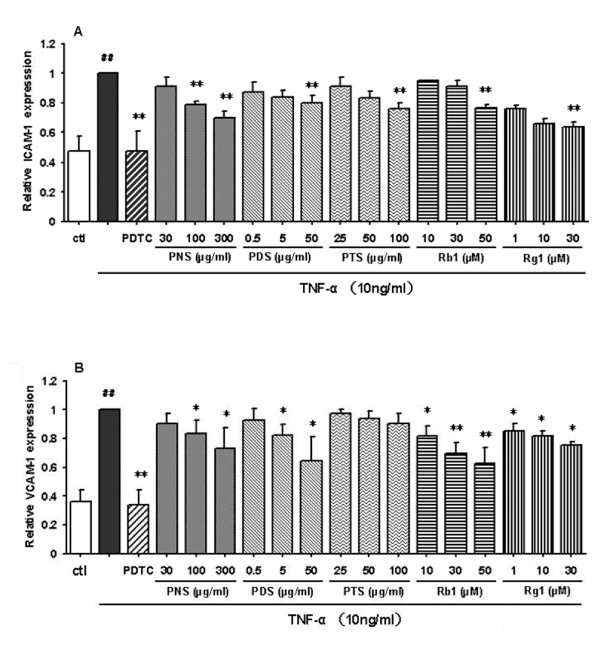
**Effects of fractions and ginsenosides from *P. notoginseng *on the surface expression of ICAM-1 (A) and VCAM-1 (B) protein in TNF-α-stimulated HCAECs**. Bars represent mean ± SD (*n *= 3). ^## ^*P *< 0.01 vs. control group; * *P *< 0.05 and ** *P *< 0.01 vs. TNF-α group.

To assess whether the fractions and ginsenosides modulate expression of TNF-α-induced adhesion molecules, we examined the effect of PNS on TNF-α-induced surface expression of ICAM-1 and VCAM-1 by immunostaining assay and cellular ELISA. The results (Figure 4) showed that both ICAM-1 and VCAM-1 were expressed at low levels on the unstimulated HCAECs. A 2-to-3-fold increase was observed upon the stimulation with TNF-α. These increases were inhibited dose-dependently by all tested samples, except that the effect of PTS on the expression of VCAM-1 was not significant at the tested concentrations of 25-100 μg/ml (Figure 4B). PDTC (10 μg/ml) could almost normalize the expression of ICAM-1 and VCAM-1 on HCAECs. Overall, the inhibitory potency of these saponin fractions on TNF-α-induced expression of the CAMs exhibited a trend similar to that of the monocyte-endothelial interaction, *ie *PDS was more effective than PTS which was more effective than PNS.

### Saponin fractions suppress the mRNA expressions of ICAM-1 and VCAM-1 in HCAECs

**Figure 5:**
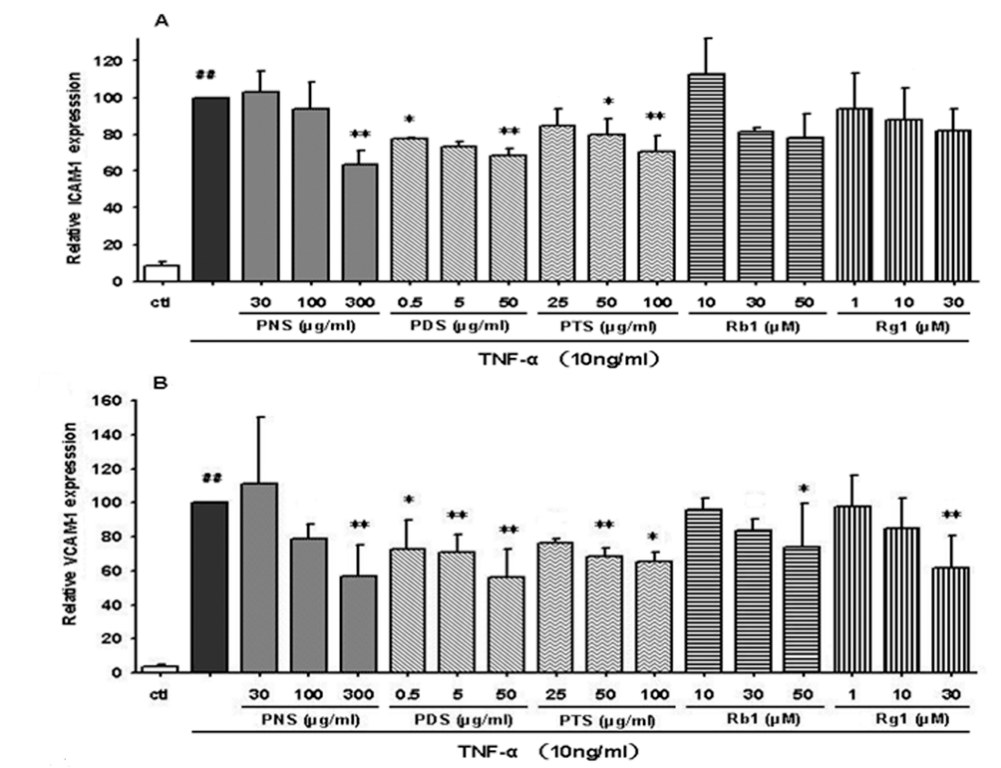
**Effects of fractions and ginsenosides from *P. notoginseng *on the mRNA expression of ICAM-1 (A) and VCAM-1 (B) in HCAECs**. Data are expressed as mean ± SD (*n *= 3), ^## ^*P *< 0.01 vs. control group; * *P *< 0.05 and ** *P *< 0.01 vs. TNF-α group.

The experiments described above demonstrated that fractions and ginsenosides inhibited ICAM-1 and VCAM-1 expression on the surface of stimulated HCAECs. It is possible that they inhibit the expression of these adhesion molecules by modulating the mRNA level. For further investigation, the total RNA of HCAECs was isolated and quantitatively assayed by qRT-PCR (Figure 5A and 5B). Pretreatment of HCAECs with the tested samples decreased the TNF-α-induced production of ICAM-1 and VCAM-1 mRNA in HCAECs. The level of inhibition of mRNA appeared to be comparable with the results of the cell surface expression experiments determined by cell ELISA.

### Saponin fractions suppress the mRNA expressions of ICAM-1 and VCAM-1 in HCD-fed rats

**Figure 6:**
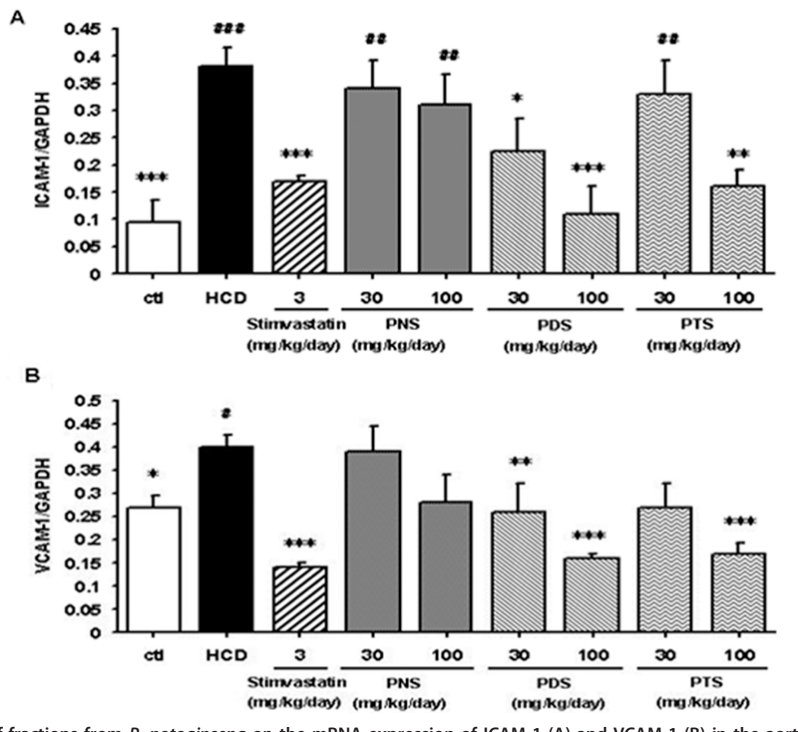
**Effects of fractions from *P. notoginseng *on the mRNA expression of ICAM-1 (A) and VCAM-1 (B) in the aortas from HCD-fed rats**. Data are expressed as mean ± SD (*n *= 6). * *P *< 0.05, ** *P *< 0.01 and *** *P *< 0.001 vs. control group. ^# ^*P *< 0.05, ^## ^*P *< 0.01 and ^### ^*P *< 0.001 vs. HCD group.

Rat thoracic aortas were isolated and the mRNA expressions of ICAM-1 and VCAM-1 were examined. Figure 6 shows that the expression of ICAM-1 and VCAM-1 mRNA in the HCD rats was higher than that in the control group. Treatment with simvastatin (3 mg/kg per day), a common used cholesterol-lowering drug, significantly inhibited the levels of ICAM-1 and VCAM-1 mRNA. Due to limited availability of pure ginsenosides for *in vivo *study, only PNS, PDS and PTS at the same dose range (30-100 mg/kg per day) were tested and compared. The trend of inhibitory action was similar to that found in the *in vitro *assays (*ie *PDS was more potent than PTS which was more potent than PNS) whereas the high dosage (100 mg/kg per day) of the PDS fraction even suppressed the up-regulated levels of ICAM-1 mRNA more efficiently than treatment with simvastatin. Treatment of the HCD rats with the saponin fractions showed differential improvements in serum lipid profile and blood vessel vasorelaxant activity (manuscript in preparation).

### Effects of the saponin fractions on the protein expression of ICAM-1 and VCAM-1

**Figure 7:**
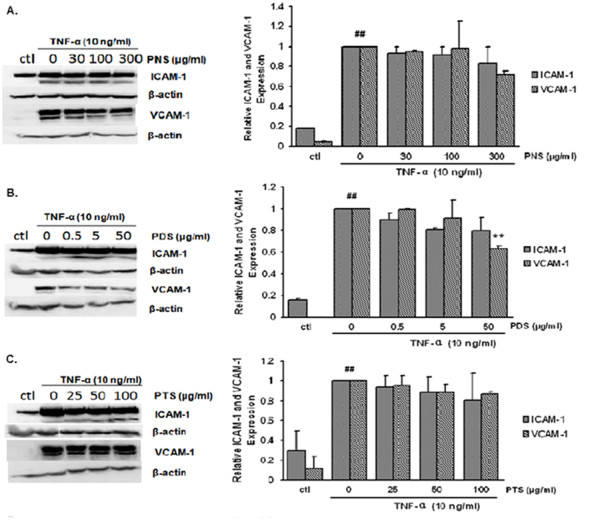
**Effects of fractions from *P. notoginseng *on the protein expression of ICAM-1 and VCAM-1 in TNF-α-stimulated HCAECs**. Samples was assayed in triplicates and normalized according to β-actin content. Data shown are expressed mean ± SD (*n *= 3). ^## ^*P *< 0.01 vs. control group; * *P *< 0.05 and ** *P *< 0.01 vs. TNF-α group.

Western blot analysis was used to investigate the effects of the saponin fractions on TNF-α-stimulated protein expressions of ICAM-1 and VCAM-1 in HCAECs. HCAECs were pretreated with various concentrations of saponin fractions for 24 hours and stimulated with TNF-α for six hours. As shown in Figure 7, relatively weak expressions of both CAMs were observed in the control group and the protein expressions of both CAMs increased significantly in TNF-α-stimulated HCAECs. Although PNS and PTS fractions showed slightly dose-dependent inhibitory effects on ICAM-1 and VCAM-1 expressions, no statistically significant difference was found. By contrast, the inhibitory effects of the PDS fraction to VCAM-1 expression (but not to ICAM-1 expression) were statistically significant (*P *= 0.0026).

### Effects of the saponin fractions on the nuclear translocation of NF-κB p65

**Figure 8:**
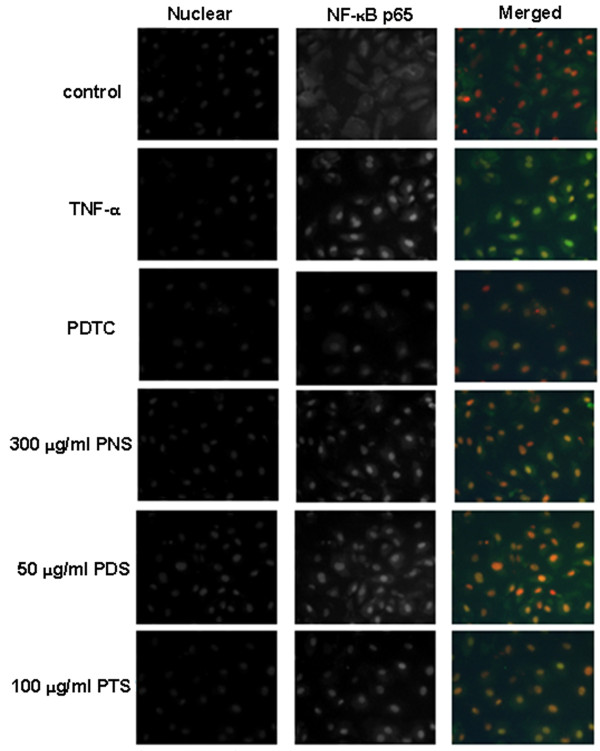
**Effects of fractions from *P. notoginseng *on NF-κB/p65 nuclear translocation**. NF-κB/p65 expression was labeled with anti-NF-κB/p65 antibody and an Alexa fluor 488-conjugated secondary antibody (green). Magnification × 400

The transcription factor NF-κB plays a key role in chronic inflammatory diseases, including atherosclerosis. Pro-inflammatory cytokines including IL-1, IL-6 and TNF-α can also induce inflammatory conditions and was regulated by nuclear factor NF-κB [15]. We used immunofluorescence microscopy to investigate the nuclear translocation of p65 as a surrogate marker for the NF-κB pathway activation. An Alexa fluor 488-conjugated secondary antibody against p65 was also used. As shown in Figure 8, the control group demonstrated that NF-κB p65 was predominantly localized in the cytoplasm. When the NF-κB pathways were activated in the TNF-α group, the translocation of NF-κB p65 into the nucleus was observed. In both the positive control and all the treatment groups, the immunofluorescent staining NF-κB p65 in cellular nucleus was less intense compared with the TNF-α group; however, we could not quantitatively compare their effects in this qualitative analysis.

## Discussion

In the present study, the actions of three saponin fractions (*ie *PNS, PDS and PTS) and two main ingredients (ginsenosides Rg_1 _and Rb_1_) on inhibiting monocyte adhesion *in vitro *and the expression of adhesion molecules were conducted and compared. We demonstrated that PNS, PDS and PTS exhibited different inhibitory activity on monocyte adhesion on the activated endothelial cells *in vitro *and the mRNA and cell surface expression of adhesion molecules, including ICAM-1 and VCAM-1, on TNF-α-activated HCAECs *in vitro*, as well as on the aorta of HCD-fed rats *in vivo*.

Many attempts have been made to establish a pivotal role of inflammation in the initial stage of atherosclerosis [2]. Elevated levels of particular cytokines, such as TNF-α and IL-6, can affect the arterial wall and cause inflammation [16-18]. In this study, TNF-α was used to activate the endothelial cells. Our data showed that three saponin fractions and two ginsenosides of *P. notoginseng *dramatically inhibited THP-1 monocyte cell adhesion to TNF-α-stimulated HCAECs in a dose-dependent manner. Among them, PDS showed the strongest inhibitory effect. These results inspired us to further investigate whether this inhibitory effect on cell-cell adhesion was caused by the down-regulation of CAMs in the HCAECs. Up-regulation of the CAMs responsible for leukocyte-endothelium interaction plays a crucial role in inflammation and atherogenesis [19]. As we expected, all tested fractions showed different inhibitory effects on the expression of ICAM-1 and VCAM-1 at both the protein and mRNA levels *in vitro*. Interestingly, the PDS fraction showed a more potent and effective anti-inflammation action on the TNF-α-activated HCAECs *in vitro *compared to the PNS and PTS fractions. More importantly, although Rb_1 _is the most abundant compound, it is not as potent as the whole PDS fraction. This finding suggests that other 20(S)-protopanaxatriol saponins in PDS (*eg *Rg_1_, Re, R_1_, Rg_2 _and Rh_1_) are likely to have the synergistic effects.

The *in vivo *effects of the three fractions on HCD-induced atherosclerosis in rats were also examined. These saponin fractions showed similar trends of different degrees of beneficial effects on improving the vasorelaxant function of blood vessels and the serum lipid profile (manuscript in preparation). Interestingly, PDS showed a stronger inhibitory effect than the PTS and PNS fractions on HCD-induced ICAM-1 and VCAM-1 mRNA levels in the rat aorta. Our *in vitro *and *in vivo *data showed PDS to be the most effective fraction in terms of inhibitory activity on the THP-1 monocyte cell adhesion to TNF-α-stimulated HCAECs as well as the expression of CAMs in the TNF-α-stimulated HCAECs and the aorta of the HCD-fed rat. However, we observed some discrepancies of the expression of VCAM-1 and ICAM-1 at both the mRNA and protein levels. CAMs is a protein family including VCAM-1, ICAM-1, E-selectin, P-selectin, PECAM-1 and mucosal addressin CAM-1 (MAdCAM-1). Only the two major members of the protein family, namely VCAM-1 and ICAM-1, were investigated in this study. It has been reported [20,21] that antioxidant agents such as PDTC and proanthocyanidin extract markedly attenuate the TNF-α-induced expression of VCAM-1 but not ICAM-1 in endothelial cells. These results provide insights into why anti-vascular inflammatory compounds elicit different transcriptional and translational regulation on CAMs.

It is well known that NF-κB controls the transcription of many genes with an established role in atherosclerosis, such as cytokines, chemokines, adhesion molecules, and macrophage infiltration [22]. NF-κB is inactive in the cytoplasm of the normal cells because it is bound to IκB. Once NF-κB is activated, degradation of IκB and the subsequent nuclear translocation of p65 as a surrogate of active NF-κB take place [23]. TNF-α as well as LPS and IL-1β may lead to the transcriptional activation of NF-κB in endothelial cells. In this study, we determined whether the protection of *P. notoginseng *saponins against vascular inflammation is associated with the regulation of the NF-κB pathway in endothelial cells. Fluorescent immunostaining experiment showed that all the saponin fractions reduced NF-κB p65 nuclear immunofluorescent staining in comparison with the TNF-α group. These results confirmed that the saponin fractions of *P. notoginseng *exhibit anti-vascular inflammatory activity probably through the inhibition of NF-κB activation. In addition, the inhibitory effects of the saponin fractions of *P. notoginseng *on adhesion molecules expressions observed in the adhesion assay, cell-ELISA, western blotting and real-time PCR analysis may be attributed to their NF-κB inhibitory actions.

Our earlier studies showed that *P. notoginseng *is unique and has high economic and therapeutic values due to its large quantity of Rg_1 _and Rb_1 _which is considerably larger than both the Asian ginseng (*Panax ginseng *C. A. Mey) and American ginseng (*Panax quinquefolius *L.) [24]. Moreover, notoginsenoside R_1 _is unique in *P. notoginseng*. Interestingly, the ratios of Rg_1_/Re and Rg_1_/Rb_1 _are higher in *P. notoginseng *compared to the other ginseng species. Our earlier studies showed that *P. notoginseng *saponins (PNS) is a potential anti-atherogenic agent [6] as well as a potential angiogenic agent for angiogenesis therapy [25]. The present study showed for the first time that the PDS fraction of *P. notoginseng *is the most active agent for the suppression of monocyte adhesion to activate endothelial cells *in vitro *and the expression of endothelial adhesion molecules *in vitro *and *in vivo*.

## Conclusion

The present study demonstrates potential anti-atherogenic effects of the saponin fractions (PNS, PDS and PTS) and major ginsenosides (Rg_1 _and Rb_1_). Among the fractions, PDS is the most effective one against TNF-α-induced cell-cell adhesion and expression of adhesion molecules *in vitro *and *in vivo*.

## Abbreviations

Apo E: apolipoprotein E; AS: atherosclerosis; CAMs: cell adhesion molecules; ELISA: enzyme-linked immunosorbent assay; GAPDH: glyceraldehyde phosphate dehydrogenase; HCAECs: human coronary artery endothelial cells; HCD: high cholesterol diet; ICAM-1: intercellular adhesion molecule-1; TNF-α: tumor necrosis factor-α; PDTC: pyrrolidine dithiocarbamate; PDS: protopanaxadiol saponin; PNS: *Panax notoginseng *saponins; PTS: protopanaxatriol saponin; VCAM-1: vascular cell adhesion molecule-1

## Competing interests

The authors declare that they have no competing interests.

## Authors' contributions

SMYL, YTW, QWZ, YHD and NY designed the study. NW, JBW and SWC carried out the experiments and data analysis. NW and JBW interpreted the data and wrote the manuscript. All authors read and approved the final version of the manuscript.
